# Genomic structural differences between cattle and River Buffalo identified through comparative genomic and transcriptomic analysis

**DOI:** 10.1016/j.dib.2018.05.015

**Published:** 2018-05-10

**Authors:** Wenli Li, Derek M. Bickhart, Luigi Ramunno, Daniela Iamartino, John L. Williams, George E. Liu

**Affiliations:** a The Cell Wall Utilization and Biology Laboratory, US Dairy Forage Research Center, USDA ARS, Madison, WI 53706, USA; b Dipartimento di Agraria, Università degli Studi di Napoli “Federico II”, via Università 100, 80055 Portici, NA, Italy; c AIA-LGS, Associazione Italiana Allevatori - Laboratorio Genetica e Servizi, Via Bergamo 292, 26100 Cremona, CR, Italy; d Davies Research Centre, School of Animal and Veterinary Sciences, University of Adelaide, Roseworthy, SA 5371, Australia; e The Animal Genomics and Improvement Laboratory, USDA ARS, Beltsville, MD, USA; f Parco Tecnologico Padano, Via Einstein, 26500 Lodi, Italy

## Abstract

Water buffalo (*Bubalus bubalis* L.) is an important livestock species worldwide. Like many other livestock species, water buffalo lacks high quality and continuous reference genome assembly, required for fine-scale comparative genomics studies. In this work, we present a dataset, which characterizes genomic differences between water buffalo genome and the extensively studied cattle (*Bos taurus* Taurus) reference genome. This data set is obtained after alignment of 14 river buffalo whole genome sequencing datasets to the cattle reference. This data set consisted of 13,444 deletion CNV regions, and 11,050 merged mobile element insertion (MEI) events within the upstream regions of annotated cattle genes. Gene expression data from cattle and buffalo were also presented for genes impacted by these regions. Public assessment of this dataset will allow for further analyses and functional annotation of genes that are potentially associated with phenotypic difference between cattle and water buffalo.

**Specifications Table**

**Table:** 

Subject area	*Biology*
More specific subject area	*Comparative genomics*
Type of data	*Tables*
How data was acquired	*Whole genome sequencing and whole transcriptome sequencing*
Data format	*Filtered and analyzed*
Experimental factors	*none*
Experimental features	*Comparative genomics between water buffalo and cattle*
Data source location	*none*
Data accessibility	Tables are with this article. Raw read data of whole genome and transcriptome sequencing were deposited to NCBI Bioprojects as the following: PRJNA350833 (https://www.ncbi.nlm.nih.gov/bioproject/?term=350833)
	PRJNA277147 (https://www.ncbi.nlm.nih.gov/bioproject/?term=277147) and PRJEB4351 (https://www.ncbi.nlm.nih.gov/bioproject/?term=PRJEB4351)
Related research article	Comparative sequence alignment reveals River Buffalo genomic structural differences compared with cattle

**Value of the data**

• This data set presents the major genomic differences between cattle and river buffalo: copy number variation deletion (CNV-deletion) and mobile element insertion (MEI).
• Genes identified in this analysis provides the basis for of further development functional assays aimed at identify genomic factors underlying phenotypic differences between cattle and buffalo.
• Structural variants and genes identified in this study will facilitate the development of resources suitable for water buffalo genomic selection.

## 1 Data

Water buffalo (*Bubalus bubalis* L.) is a significant livestock species worldwide with high economic importance [1]. This study sought to characterize differences in gene content, regulation and structure between taurine cattle (2*n* = 60) and river buffalo (2*n* = 50) (one extant type of water buffalo) using the extensively annotated UMD3.1 cattle reference genome as a basis for comparisons. Using 14 WGS datasets from river buffalo, we identified 13,444 deletion CNV regions (Supplemental Table 1) in river buffalo, but not identified in cattle. We also presented 11,050 merged mobile element insertion (MEI) events (Supplemental Table 2) in river buffalo, out of which, 568 are within the upstream regions of annotated cattle genes. Furthermore, our tissue transcriptomics analysis provided expression profiles of genes impacted by MEI (Supplemental Tables 3–6) and CNV (Supplemental Table 7) events identified in this study. This data provides the genomic coordinates of identified CNV-deletions and MEI events. Additionally, normalized read counts of impacted genes, along with the adjusted p-values of statistical analysis are presented (Supplemental Tables 3–6).

## 2 Experimental design, materials and methods

### 2.1 Data used and experimental design

Genomic DNA samples from river buffalo were provided by the International Water Buffalo Genome Consortium. Sequence data was generated at the USDA Agricultural Research Service (Beltsville) on an Illumina Genome Analyzer II. All sequencing data were submitted to NCBI (accession #PRJNA350833). Genomic sequencing reads from cattle were deposited to NCBI (accession #PRJNA277147). For whole transcriptome sequencing data, raw reads of river buffalo tissue transcriptomics were deposited to NCBI (accession #PRJEB4351). For cattle, we used RNA-seq data from the Angus breed (accession #PRJNA311009).

This study used the extensively annotated UMD3.1 cattle reference genome as a basis for comparisons between river buffalo and cattle, by aligning whole genome shotgun sequencing reads from river buffalo to the cattle reference genome. To identify river buffalo specific, genomic variants, CNV, SNP and MEI calls resulting from the cattle WGS reads were used as a background filter to remove variant sites previously identified in cattle from the river buffalo dataset.

### 2.2 Structural variant calling

To detect mobile element insertions (MEIs), RAPTR-SV [2] version 0.0.14 (run with default parameters) and RepeatMasker (http://www.repeatmasker.org/) were used. We selectively focused on trans-chromosomal read pair alignments from RATPR-SV's preprocess divet file format. RepeatMasker generated tabular output from the cattle reference genome was used to determine candidate repetitive origins of trans-chromosomal reads. Using a custom Java program that selectively clusters trans-chromosomal read pairs and intersects them with repetitive elements (https://github.com/njdbickhart/MEIDivetID), only discordant reads unlikely to consist of misaligned repetitive elements were considered in this analysis. To ensure that trans-chromosomal repetitive reads were not simply misalignments of local repeats to the wrong chromosome, the program searched for the nearest repetitive element of the same class (as determined by RepeatMasker) within 1 kb of the anchor read fragment. If none were found, the event was output as a putative MEI near the anchor read position, with the true event assumed to be downstream of the forward orientation of the anchor read, and within a distance close to the sequence library average insert size. Bedtools suite [6] was to identify genes impacted by MEI events. Genes and their promoter regions were included to identify intersections.

To identify copy number variations, cn.mops [3] version 3.5 and JaRMS [4], a Java language port of the CNVnator software package [5] was used. The Bedtools suite [6] was used to find consensus calls between JaRMS and cn.mops CNV and custom perl scripts (https://github.com/njdbickhart/perl_toolchain). CNV deletions shared by both JaRMS and cn.mops were further intersected with cattle gene coordinates.

### 2.3 Comparative gene expression analysis between cattle and river buffalo

RNA-sequencing reads from river buffalo (NCBI, PRJEB4351) and the Angus breed of cattle (NCBI, PRJNA311009) were used to compare the expression differences of genes impacted by MEI and CNV-deletions. For MEI-impacted genes, RNA-seq data from liver and muscle were used. For CNV-deletion impacted genes, analyses were performed for all the tissues for which we had RNA sequencing data. To avoid potential quantification bias introduced by sequencing depth, gene-level, raw read counts obtained from STAR [7] were normalized/divided by a “per million reads” factor (obtained by dividing the total # of raw read counts by 1,000,000). Normalized read counts produced by the above steps were then used for gene expression comparisons between cattle and river buffalo. SAM (significant analysis of microarrays) [8], [9], [10], [11] was used to calculate statistical significance of gene expression differences in river buffalo compared to cattle (< 0.05, *q*-value cutoff used).
